# Mask Attention-SRGAN for Mobile Sensing Networks

**DOI:** 10.3390/s21175973

**Published:** 2021-09-06

**Authors:** Chi-En Huang, Ching-Chun Chang, Yung-Hui Li

**Affiliations:** 1 AI Research Center, Hon Hai Research Institute, Taipei 114699, Taiwan; a3285556aa@gmail.com; 2 Department of Computer Science, University of Warwick, Coventry CV4 7AL, UK; ching-chun.chang@warwickgrad.net

**Keywords:** super-resolution, attention mechanism, Generative Adversarial Network, biometric authentication, biometric identification, mobile sensing network

## Abstract

Biometrics has been shown to be an effective solution for the identity recognition problem, and iris recognition, as well as face recognition, are accurate biometric modalities, among others. The higher resolution inside the crucial region reveals details of the physiological characteristics which provides discriminative information to achieve extremely high recognition rate. Due to the growing needs for the IoT device in various applications, the image sensor is gradually integrated in the IoT device to decrease the cost, and low-cost image sensors may be preferable than high-cost ones. However, low-cost image sensors may not satisfy the minimum requirement of the resolution, which definitely leads to the decrease of the recognition accuracy. Therefore, how to maintain high accuracy for biometric systems without using expensive high-cost image sensors in mobile sensing networks becomes an interesting and important issue. In this paper, we proposed MA-SRGAN, a single image super-resolution (SISR) algorithm, based on the mask-attention mechanism used in Generative Adversarial Network (GAN). We modified the latest state-of-the-art (nESRGAN+) in the GAN-based SR model by adding an extra part of a discriminator with an additional loss term to force the GAN to pay more attention within the region of interest (ROI). The experiments were performed on the CASIA-Thousand-v4 dataset and the Celeb Attribute dataset. The experimental results show that the proposed method successfully learns the details of features inside the crucial region by enhancing the recognition accuracies after image super-resolution (SR).

## 1. Introduction

Biometrics has been widely used in the various recognition tasks for many of the essential services, such as large-scale identity management, fugitive hunting, and immigration checkup [1,2,3]. For those applications, how to maintain the high quality of the input image is crucial, which heavily depends on several of the optical factors, such as the focal length, field of view, depth of focus, and the combination of them. On the sensor side, higher resolution is desirable for most Internet of Things (IoT) applications. Additional hardware or more expensive sensors are the common solutions for such requirements, which leads to higher cost. It also may encounter the physical limitations of the device. For example, increasing the density of the pixel in a unit area is not always possible if the size of the sensor chip has to be small and the high density of sensors also causes a decrease of the number of photons that can be sensed per unit area, leading to low intensity of the captured image.

One of the most dynamic and exciting developments in information and communications technology is the advent of the IoT. Some recognition tasks may be deployed on the IoT device to decrease the cost and increase the capability of concurrency at the same time [4]. However, it is hard to preserve the high-resolution (HR) image on such low-cost devices due to the limitation of the hardware and the storage space or bandwidth of sensing networks. For biometrics-oriented IoT devices, this is not good news since there is a standard requirement of spatial sampling ratio in both of the iris image and the face image which were proposed by the ISO/IEC 29794-6 [5] and the ISO/IEC 19794-5 [6], as shown in Table 1. So, the IoT devices may not satisfy the minimum requirements of these standards for biometrics. Therefore, it is desirable if the resolution of the captured biometric image can be enhanced using computer vision techniques. Such technique is called SR and it has been developed over decades [7]. According to the mentioned framework, the advantage of applying the SR technique to biometrics recognition is that the recognition accuracy will be preserved with the low-resolution input image. In such a way, we can achieve the high recognition rate with the low-cost IoT device.

The SR technique can be classified into two categories: spatial domain SR and frequency domain SR. The methods in the spatial domain SR can be further divided into two sub-classes: single-image SR (SISR) and multi-image SR (MISR). The methods based on SISR attempt to reconstruct the image back into its HR counterpart with only one given low-resolution (LR) image. In the image acquisition phase, IoT devices are often limited to lower resolution, so SISR may be a preferable framework for the IoT applications.

GAN as one of the most famous framework of deep learning method has demonstrated potential capabilities in many fields [8]. Specifically, the SR technique based on GAN methods (SR-GANs) achieves outstanding performance for the tasks of computer vision. The SR-GAN enhance the quality of LR images via the various loss functions, which have been proposed to improve the image quality from a different perspective. However, most of the loss functions used in the previous work of GAN may not consider the regions of interests (ROI) in the LR images. In recent years, several SR methods proposed the attention mechanism attempt to improve their work. Although some attention-based model certainly performed better than the previous works on the general metrics, most of the metrics may not completely reflect the evaluation of human vision perception. Moreover, the generated SR images, which have a higher score in the metric, makes virtually no difference to the visual perception compared to the previous works.

**Figure 1:**
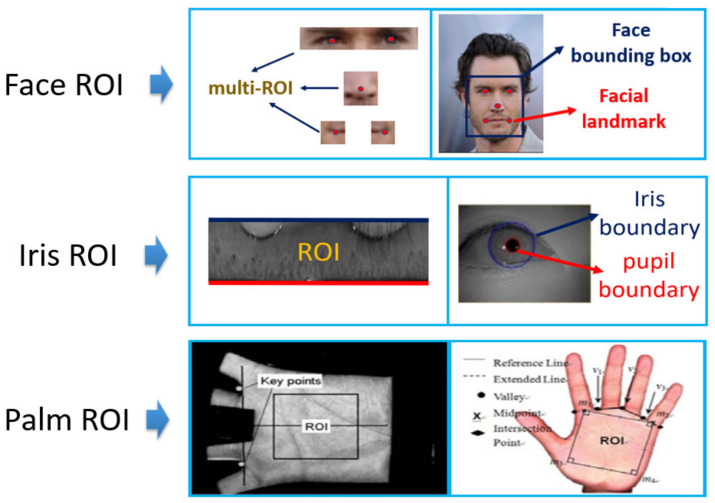
Various ROIs in biometrics.

However, the attention mechanism may still be useful in the other applications of SR, because what is pleasing to human visual perception may not necessarily be good for achieving high accuracy for classification-related tasks. Indeed, there are specific parts (ROI) for biometric images, like iris region or facial landmarks, which contain very discriminative information compared to another region in the same picture. The various ROIs for some biometric applications are shown in Figure 1.

In this work, we propose the mask-based attention approach to force GAN to focus on the appearance difference of ROI between the super-resolved image and the corresponding HR image. According to the feedback of discriminator, the generator will be trained to learn how to recover more detailed information inside ROI while learning structure and texture information between LR and HR images.

To summarize, our main contributions are as follows:

1. We propose MA-SRGAN, which is a GAN-based SR framework using attention mechanism. It gives attention to user-defined ROI region, which is a novel idea for SRGAN framework.
2. In the training procedure, we add the attention loss to the state-of-the-art nESRGAN+. In this way, the generator is forced to pay more attention to ROI. The super-resolved image will be much more useful for classification-oriented task like biometric recognition.
3. We propose a new perspective for the quantitative evaluation of the effectiveness of SR, according to the needs of the downstream tasks. In this paper we mainly care about how to use SR to enhance the input image for biometric recognition in a mobile sensing network. Therefore, the quantitative evaluation metric of SR we use will be the domain-specific metric (biometric recognition domain), such as Equal Error Rate (EER). Furthermore, the more generic metrics, such as Peak Signal-to-Noise Ratio (PSNR) and Structural Similarity Index (SSIM), will be ignored in the domain-specific work in order to fairly measure the effectiveness and usefulness of the SR for downstream tasks.

We obtain a series of comparable results with higher verification rate. On Celeb-Attribute, it is able to achieve 89.75% Verification Rate (VR) with 5% False Accept Rate (FAR), and the EER is 6.23%. Moreover, on the CASIA-Thousand-v4, it is able to achieve 92.23% VR with 1% FAR and attains comparable performance with 2.3% EER. Both these error rates are lower than that of the state-of-the-art GAN-based SR model.

## 2. Literature Review

The approaches for SISR can be mainly categorized into three kinds: interpolation-based methods, reconstruction-based methods, and rule-based methods [9]. The deep learning approach has further surpassed the former methods in recent years. Therefore, we review deeplearning based approaches in this section.

Dong et al. [10,11] proposed SRCNN as the first work of a deep-learning approach. It made use of the convolution network to learn the non-linear mapping from LR to HR in an end-to-end manner, and to achieve superior performance against previous works. After that, with the various networks that were proposed, the later development of SR also made the most use of those structures. For example, Kim et al. [12] proposed VDSR which made use of the residual learning to fit the deeper network. With similar performance, DRCN [13] exploited deep recursive networks by combining intermediary results. Inspired by the Res-Net [14], VDSR, and DRCN, Ying Tai et al. proposed the DRRN [15] to integrate the previous method into residual units with slight adjustments. Moreover, Tong et al. [16] proposed the SRDenseNet which made use of the dense connected convolutional networks with the single skip connection to increase the combination of the features at a different level. Base on the backbone of the DenseNet [17], the Yulun Zhang et al. [18] further proposed the RDN, which combined the dense connected convolutional networks with the residual connections, and exploited the hierarchical features from different convolution layers and fused them to present on the generated image.

As the pioneer of the GAN-based framework in solving SR, Christian Ledig et al. [19] proposed SRGAN with the perceptual loss [20,21] as well as the adversarial loss. In EDSR [22] and MDSR (which was a multiple scale factors version of EDSR), the authors eliminated the unnecessary part to further achieve the performance of state-of-the-art SR under the metric of PSNR. In the GAN-based framework, the perceptual loss allows the generated image more suitable for the human visual system. These images are visually more convincing despite having a lower score on traditional metrics of quantitative measures, like PSNR and SSIM.

ESRGAN [23], as its name implies, enhances SRGAN. It introduced a new block named RRDB in the generator. The RRDB achieved a higher capacity by adding the residual connection in the main path of Residual Dense Block used in SRGAN. The discriminator used the relativistic average loss [24] to evaluate “whether one image is more realistic than the other in the average of expectation”, and vice versa, which forced the discriminator not only to focus on the fake image but also to learn more information about the real one. In the part of feature extraction, the extracted features by VGG [25] were taken before activation rather than after, as in SRGAN. In order to train a deeper network with higher stability, the several tricks, such as residual learning and BN layer removal, were applied.

Furthermore, Nathanael Carraz Rakotonirina et al. [26] proposed nESRGAN+, which was the enhancement of ESRGAN, by placing the RRDB into the RRDRB via further adding the concatenation path in the inner dense block, which will increase the network capacity. The trick of giving finer details in the high-level aspects meant adding the Gaussian noises on the main path of the RRDRB structure.

Recently, the attention mechanism was used in the SR and was gradually integrated into the GAN. Yulun Zhang et al. [27] proposed the RCAN which made use of the residual backbone with the channel attention mechanism to adaptively rescale channel-wise features by considering interdependencies among channels. Base on the attention mechanism on the channels, Tao Dai et al. [28] further proposed the RBAN framework, which consisted of two types of attention modules in the residual blocks to exploit the vital feature from the spatial and channel dimensions. On the other hand, Deokyun Kim et al. [29] proposed the novel facial attention loss to focus on restoring the facial attributes in greater detail, while the FAN network was also proposed to extract the heatmap value to present the ROI of facial landmarks.

In the biometric applications, the usefulness of the image may not equal the image quality evaluated by the human visual system or some metrics based on global image quality (like PSNR or SSIM). For example, for iris recognition, the details in iris texture region are much more important than the details in another region in the picture. This factor should be taken into account when we want to design a novel SR algorithm for biometrics-related applications. The present work attempts to propose a novel deep learning architecture for an SR task, specifically useful for biometric recognition in sensing networks.

## 3. Materials and Methods

**Figure 2:**
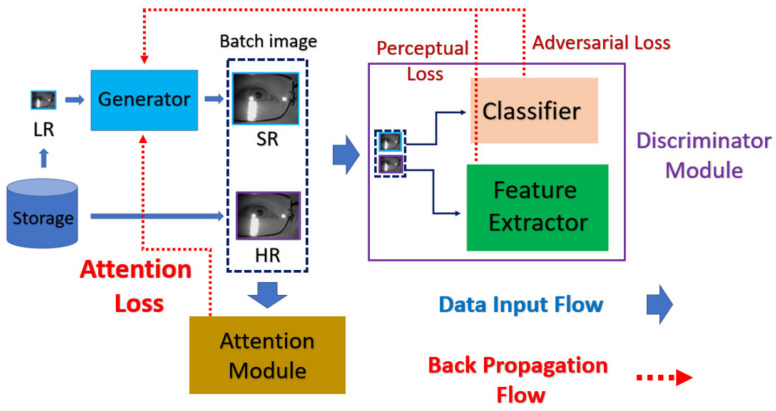
The flowchart of the MA-SRGAN.

The proposed method attempts to enhance the image quality within ROI by the attention mechanism in the spatial domain. In this section, we first describe the mask labeling for biometrics to mark the ROI, and then discuss the network structure as well as the corresponding loss function. Lastly, we describe the proposed attention module as well as the novel loss function of the generator. The flowchart of the proposed method is shown in Figure 2.

### 3.1. Mask Labeling for Biometrics

**Figure 3:**
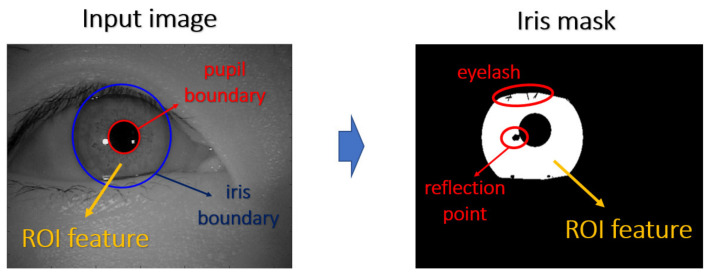
Iris mask labeling for biometrics.

**Figure 4:**
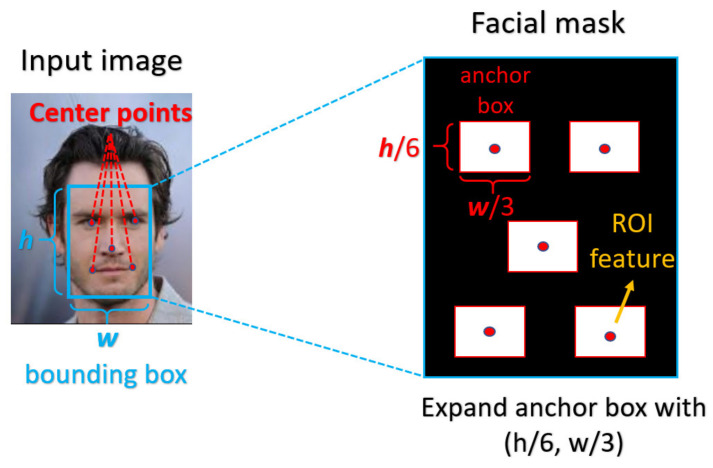
Face mask labeling for biometrics.

In order to precisely indicate the ROI in the input image, we designed each mask according to the corresponding domain knowledge to extract the discriminating features. For the iris mask, most of the features are located at the region between the iris boundary and the pupil boundary, and the noise of the image, such as eyelash and reflection points, will be further excluded from ROI. For the facial mask, we use the facial landmarks as the center points of the ROI anchor box, and the anchor boxes have a suitable shape in accordance with the aspect ratio of the corresponding face bounding box. From a practical view, the simple five-point landmarks, which are the coordinates of eyes, nose, and mouth corners, will be used as the center points during our experiment. A pictorial example of the corresponding mask is depicted in Figure 3 and Figure 4, respectively. In our proposed method, the mask will be a given input to the attention mechanism. In the training phase, we took the manually labeled mask as the input to guarantee that the ROI is perfectly accurate, while the labeled masks will also be used during the test phase.

### 3.2. Attention Mechanism on SR-GAN

#### 3.2.1. SR-GAN Network Architecture

**Figure 5:**
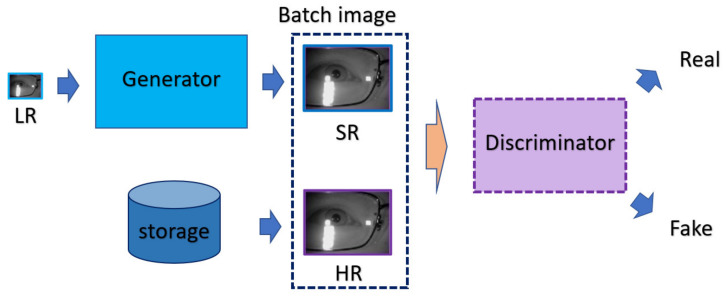
The training process of nESRGAN+.

nESRGAN+ is the well-known model in the field of SRGAN, which is composed of the generative model G and the discriminative model D. As the general framework of GAN, the G attempts to fool the D via generated SR image from the given LR counterpart, and the D attempts to perform discrimination based on the subtle difference between the given (HR, SR) pairs, as illustrated in Figure 5.

In the framework, G is primarily made up of the basic element RRDRB, which is the dense block with the residual connection repeated in different levels of the network.

**Figure 6:**
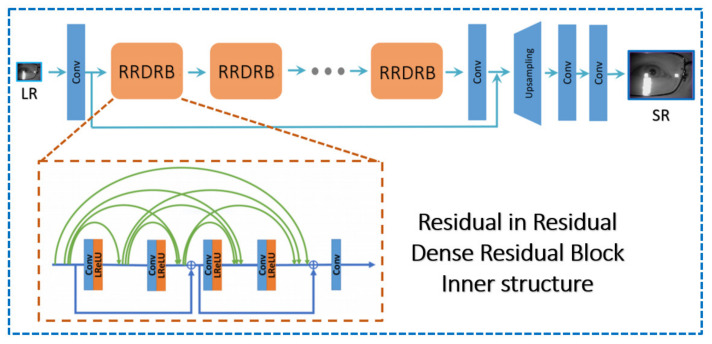
The network structure of the generator in nESRGAN+.

**Figure 7:**
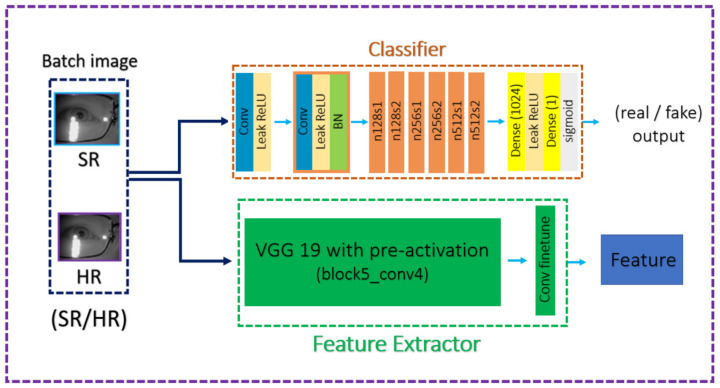
The network structure of the discriminator in nESRGAN+.

Similarly, D is composed of the VGG-based module and the deep convolution module. The former classifies the image on the perceptual level, and the latter works toward minimizing PSNR. For more details for the network structure, please refer to Figure 6 and Figure 7.

On the other hand, G and D play the following two-player minimax game with value function $V\left(G,\;D\right)$ as described in Equation (1).

(1) $$\min_{G}\max_{D}V\left(G,\;D\right)\;=\;E_{hr\sim P_{HR\_img}}\left[\log\left(D\left(hr\right)\right)\right]\;+\;E_{lr\sim P_{LR\_img}}\left[\log\left(1-D\left(G\left(lr\right)\right)\right)\right],$$

In order to generate more realistic image, the loss function of G further combined with the perceptual loss, adversarial loss, and pixel-wise $L_{1}$ loss with the different weights. Moreover, the corresponding loss function of the novel proposed attention module will further extend the loss function terms, and the overall loss function of generator is defined as Equation (2).

(2) $$L_{G}=L_{percep}^{befVGG}+\lambda L_{adv}^{Ra}+\eta L_{pixel}^{L1}+\gamma Atten_{loss}$$

where λ, η, γ are weighting coefficients.

(3) $$L_{percep}^{befVGG}=-E_{\begin{array}{c} hr\sim P_{HR\_img}\\ lr\sim P_{LR\_img} \end{array}}∥\varphi_{i,j}\left(hr\right)\;-\;\varphi_{i,j}\left(G\left(lr\right)\right){∥}_{2},$$

(4) $$L_{adv}^{Ra}=-E_{\begin{array}{c} hr\sim P_{HR\_img}\\ sr\sim G\left(lr\right) \end{array}}\left[\log\left(1-D_{Ra}\left(hr,\;sr\right)\right)\right]\;-\;E_{\begin{array}{c} hr\sim P_{HR\_img}\\ sr\sim G\left(lr\right) \end{array}}\left[\log\left(D_{Ra}\left(sr,\;hr\right)\right)\right],$$

(5) $$L_{pixel}^{L1}=-E_{lr\sim P_{LR\_img}}∥G\left(lr\right)\;-\;y{∥}_{1},$$

In the Equation (3), $\varphi_{i,j}$ denotes the VGG-extractor, which extracts the feature from i-th layer before the j-th activation. In the Equation (4), $D_{Ra}$ denotes the relativistic discriminator [15].

#### 3.2.2. Loss Function of Attention Mechanism

Although attention has already been applied to the various issues of computer vision [20,21,22], it is barely discussed in the field of SR, since each pixel in the SR image is equivalently important in the original SR problem. In this paper, we introduce the idea of attention mechanism in domain-specific SR issue, so that the intentionally recovered detail will be preferable to the final goal of the desired problem (for example, higher accuracy in biometric recognition problems). The source to indicate the direction of generating the SR image is mainly derived from the judgement of the discriminator. So, we place the attention mechanism on the discriminator, which allows the generator to recover ROI texture indirectly.

**Figure 8:**
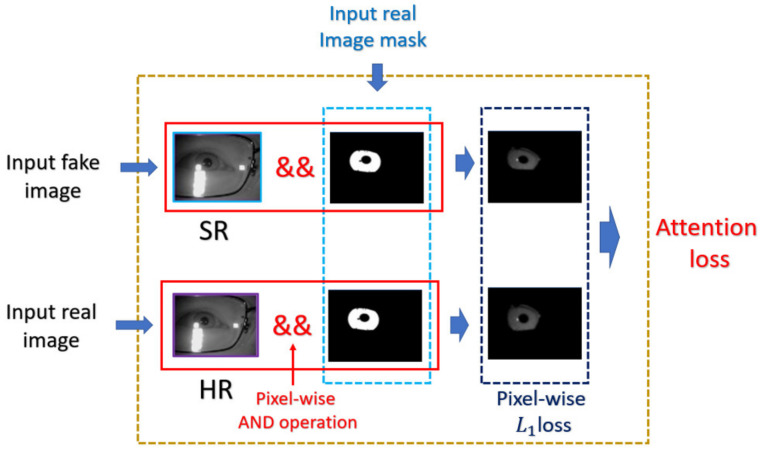
Attention module and the corresponding loss in the proposed network.

In this paper, we propose the novel component of the discriminator named mask attention, which is derived from the previous work of nESRGAN+. The mask attention module takes the prepared boolean mask as input, then focuses on the ROI with simply executing the element-wise multiplication between the mask. The mentioned attention module is illustrated as Figure 8, and the corresponding novel loss function of the generator as Equation (6)

(6) $$Atten_{loss}=E_{\begin{array}{c} hr\sim P_{HR\_img}\\ lr\sim P_{LR\_img} \end{array}}\;ROI_{part}\left(hr\right)\;-\;ROI_{part}\left(lr\right)$$

(7) $$ROI_{part\left(Img\right)}=\;\sum_{i=1}^{W}\sum_{j=1}^{H}\left|Img_{i,\;j}*msk_{i,j}\right|,\;msk_{i,j}\in \left\{0,\;1\right\}$$

In the Equation (7), the image with high-resolution shape denotes Img and the corresponding width and height of the image denote W, H, respectively. Note that the super-resolution image has exactly same shape as the high-resolution image. Besides, the binary ROI mask denotes msk, which has the same shape as the high-resolution image.

The benefit of the mask attention is that the discriminator can learn to decide the important features, and the generator is also forced to learn the correspondence between the LR and HR counterparts within ROI, which results in images of superior quality, satisfying both human perception and the requirement of biometric recognition.

## 4. Experiments

The proposed MA-SRGAN can be used in the various fields of computer vision tasks, for example, biometric authentication or medical image enhancement. In this section, we will take the iris recognition as well as the face recognition as cases for the Proof of Concept (PoC) to go through the experiment, and demonstrate the effectiveness of the proposed method. We first describe the process of biometrics and then discuss the corresponding dataset used in the experiment. After that, we describe the training details and the experimental procedure. Lastly, the experimental results will be presented.

### 4.1. Domain Knowledge of Biometrics

#### 4.1.1. Common Procedure of Biometrics

**Figure 9:**
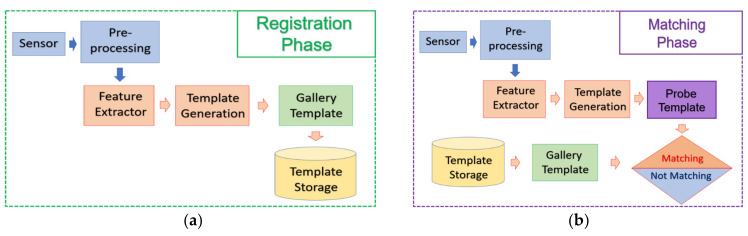
Common Phase in biometrics: (**a**) registration phase; and (**b**) matching phase.

Although most of the biometrics have their independent procedure to pre-process the input data, there exists a general procedure in terms of biometric template registration and matching. The whole procedure is illustrated in Figure 9.

In the feature registration phase, the users will enroll their biometric trait and the acquired images will be processed by the recognition system to extract the feature. The extracted feature will be saved into the template storage.

In the feature matching phase, the biometric traits of the users will be acquired by the biometric sensor and the acquired image will be processed by the recognition system to extract the feature. The extracted feature will be compared against all other features stored in the template storage and a similarity (or distance) value will be computed for every possible pair. The result of identification or authentication can be determined based on these similarity values.

Next, we further introduce the feature extraction process for iris recognition and face recognition and then discuss how to embed the proposed SR method into these processes to enhance the recognition results.

#### 4.1.2. Iris Recognition Process

According to the framework proposed by Daugman [30,31,32,33,34], the process of iris recognition can be divided into four stages: image acquisition, image preprocessing, feature extraction, and feature matching.

At first step, the eye image is captured by a Near-infrared (NIR) camera sensor which is fine-tuned for iris image capturing, since most rich structures of the iris, such as the cratered surface of the moon, appear in the NIR band. The preprocessing, including iris segmentation and iris coordinate transformation, is executed so that the iris texture (which is the ROI for iris recognition purpose) in the original image will be transformed into the polar coordinate system, resulting in another representation of the iris image in a rectangular shape. After that, the iris features will be extracted and converted into the vector of a binary string—these are called iris codes. The feature is compared with the already stored iris templates. In our experiments, the Harr-wavelet based feature extraction method was used for feature extraction of the iris images. Note that such method is able to extract the feature in various resolution and generating the feature template within the same dimension, so that the template is able to further perform the cross-resolution matching.

**Figure 10:**
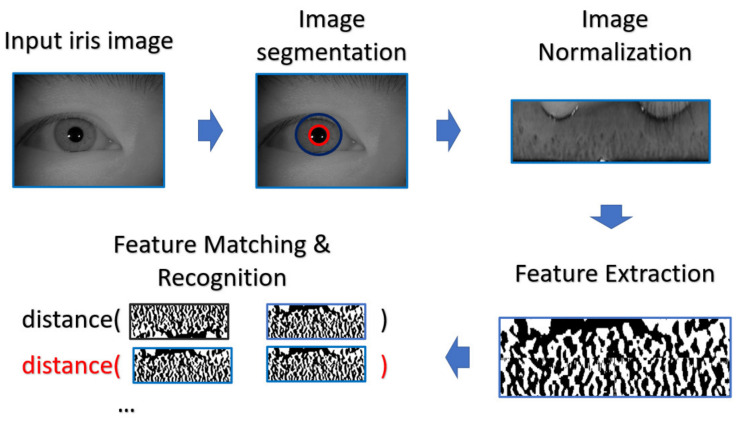
The flowchart of iris recognition in our experiment.

During the matching phase, the probe iris code is matched against all iris codes in the enrolled database. This matching is performed by computing the Hamming distance (HD) between the two iris codes. In our experiments, the threshold value of HD for acceptance or rejection is selected by considering the best EER. The overall process is depicted in Figure 10.

#### 4.1.3. Face Recognition Using Deep Learning

**Figure 11:**
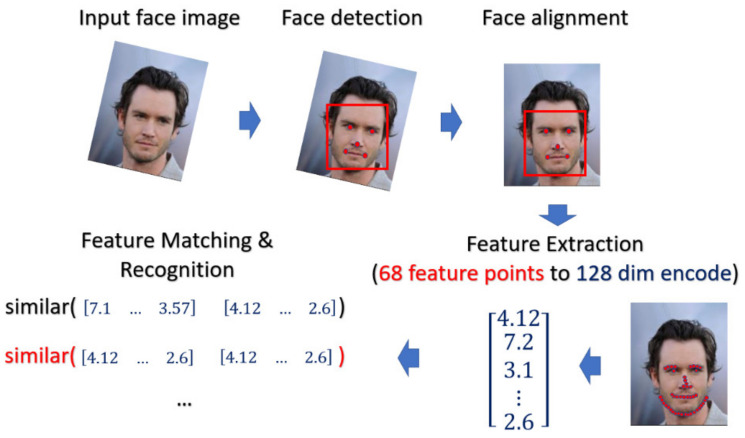
The flowchart of face recognition in our experiment.

For face recognition, the face image is captured by the optical sensor, which can be either RGB sensor or NIR sensor. The preprocessing including face detection and face alignment is executed so that the location of face can be detected and the input face can be properly aligned. After that, depending on which model or classifier is chosen for the recognition, there exist different ways for feature extraction for face biometrics. In our experiment, we adopt a deep-learning based model. We applied Dlib library [35] on the aligned face to extract the feature and encode it into the 128 dimensional vector and the encoded value will be normalized into the range [0, 1]. The feature extractor in Dlib applied a very deep residual network as the backbone to extract the face feature as well as to encode the facial identities. In the matching phase, the probe face code is matched against all the face templates in the enrolled database by computing the distance between the two face codes. In this paper, the threshold value of the distance is selected by considering the best EER. The overall process is depicted in Figure 11.

### 4.2. Dataset

The dataset we used for the iris recognition and for the face recognition are CASIA-Thousand-v4 [36] and Large-scale CelebFaces Attributes [37], respectively. The former dataset is the biggest public iris dataset in the world currently, and the latter one is often used as the benchmark for face recognition.

The CASIA dataset contains a total of 20,000 images with 2000 classes, and each class contains 10 eye images with resolution 640 × 480. We created the iris mask manually for every image in this dataset. On the other hand, the CelebA dataset contains 202,599 images with 10,177 classes originally. However, each class contains various numbers of images. Due to the number of images contained in each class being unbalanced, we preprocess the dataset so that each class contains 20 images with resolution 160 × 200 and the resulting number of classes used for experiments is 6000. Besides, the data augmentation is performed on both iris images and face images through random horizontal flips. The above information is shown in Table 2.

### 4.3. Training Details and Parameters

The LR images are obtained by the bicubic down-sampling with a scaling factor of four from the HR images. The size of the mini-batch for iris generation is 4 due to the memory limitation, and the size of the mini-batch for face generation is 16. The following setting for the training uses the same specification for both iris and face generation.

Since the proposed MA-SRGAN is based on the backbone of nESRGAN+, the most of the parameters are still unchanged to achieve the optimized setting. At first, we also make use of the PSNR-oriented pre-trained model to initialize the generator. The values of λ and η are set to be the value described in the original nESRGAN+ paper [26] (λ = 5 × 10^−3^, η = 1 × 10^−2^), and the value of γ is set the same as η. The learning rate is set to 1 × 10^−4^, and the model is optimized using Adam with β_1_ = 0.5 and β_2_ = 0.999 with training 10,000 epochs. The trained model is the one with the three-blocks generator. The implementation is done with Keras based on the TensorFlow backend, and trained with four NVIDIA GeForce GTX-1080 GPUs.

### 4.4. Experimental Design

The dataset used in the experiment is split into the training and the evaluation set and the evaluation set is further divided into the probe set and the gallery set. In the training phase, half of the classes will be used to train the model, and the sample will be randomly picked without duplicate in the dataset. In the evaluation phase, we attempt to simulate the practical situation of biometric recognition, where the system (in most cases) registers the higher quality images during the enrollment process. So, the gallery set contains the high-resolution image as the ground truth, while the probe set contains either the low-resolution (LR) image or the super-resolved (SR) images generated from the LR images.

**Figure 12:**
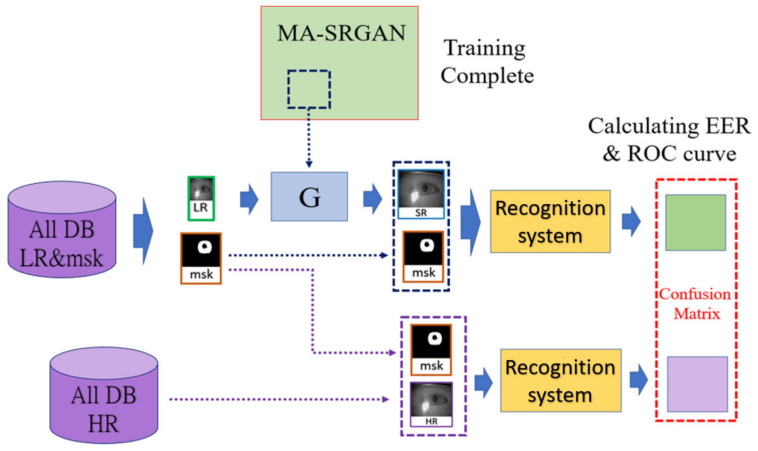
The procedure for SR-enhanced biometric recognition, as described in Section 4.4.

At first, we will compute the recognition performance with the probe images with the high resolution, which is the original resolution of the image as the Section 4.2 described, and with the low resolution, which is down-sampled to 1/4 from the high resolution. For SR performance comparison, we will compare the proposed MA-SRGAN with some recent deep-learning based methods. Figure 12 illustrates the entire procedure of the experiment.

### 4.5. Experimental Result

For SR performance evaluation in this paper, instead of using the traditional metrics, such as PSNR or SSIM, we measured the SR performance by evaluating how much the performance of the downstream task can be enhanced. The traditional metric like PSNR or SSIM measure the similarity between the HR and SR image globally, which means it treats the pixels inside and outside ROI with equal weight. However, in the proposed method, the model is forced to learn the correspondence between HR and SR images inside ROI, and such mechanism is particularly useful for specific downstream tasks, like biometric recognition. Therefore, in order to fairly measure the performance of the proposed method, we evaluate the SR performance by looking at how much improvement it brings to the biometric recognition accuracy. Typically, the biometric recognition accuracy can be compared using ROC curve, EER, Fisher ratio between the authentic and imposter distribution, and verification rate given a predefined FAR. Therefore, we will use these metrics to compare the SR performance between the proposed method and other baseline methods. The following section will describe the effect of the proposed SR method for the biometrics in terms of the iris recognition as well as the face recognition.

#### 4.5.1. Downstream Task: Iris Recognition

**Figure 13:**
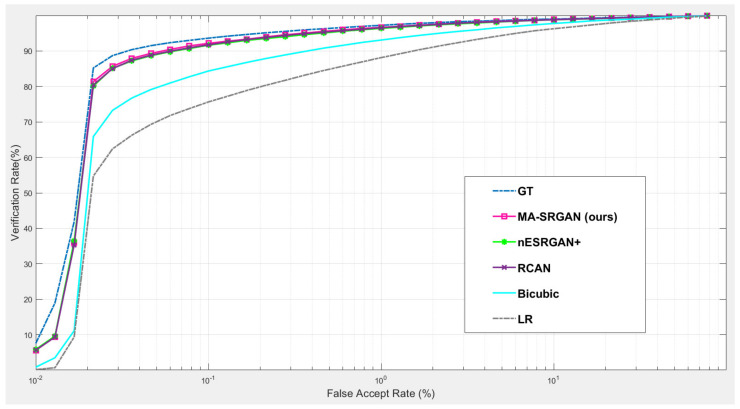
The comparison of the ROC curves of iris recognition based on input images enhanced by the various SR models using CASIA dataset.

**Figure 14:**
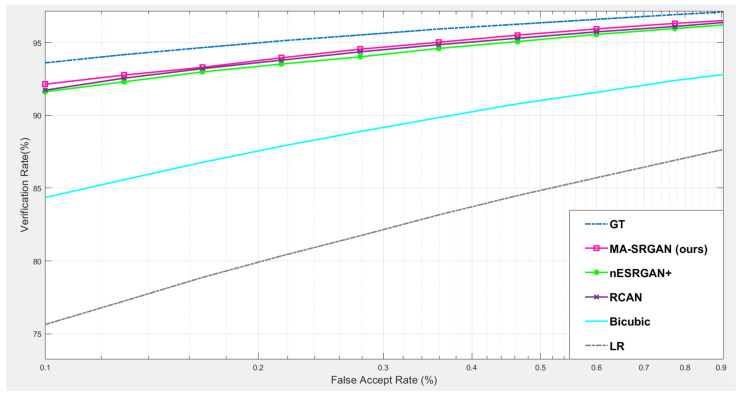
The comparison of ROC curves between nESRGAN+ (current SOTA) and MA-SRGAN (SR/wMsk) on the CASIA dataset.

For iris recognition experiments, we observe that the proposed MA-SRGAN performs better than the most of super resolution methods in terms of EER as illustrated in Figure 13. In the further comparison between MA-SRGAN and nESRGAN+, which is the latest state-of-the-art (SOTA) of SRGAN, our proposed method has better performance than nESRGAN+ in the situation of low FAR, and achieves the lower EER with 2.3%, as described in the Figure 14 and Table 3, respectively. The ground truth (HR) images achieve an EER of 2.072%.

This highlights the benefits of using the attention module in the discriminator network. It can be observed that the images reconstructed by our model present more detailed information about the distinguishable part of the iris, which in turn, enhances the iris recognition accuracy. It shows the proposed method enable the deep model to learn useful details inside ROI.

#### 4.5.2. Downstream Task: Face Recognition

**Figure 15:**
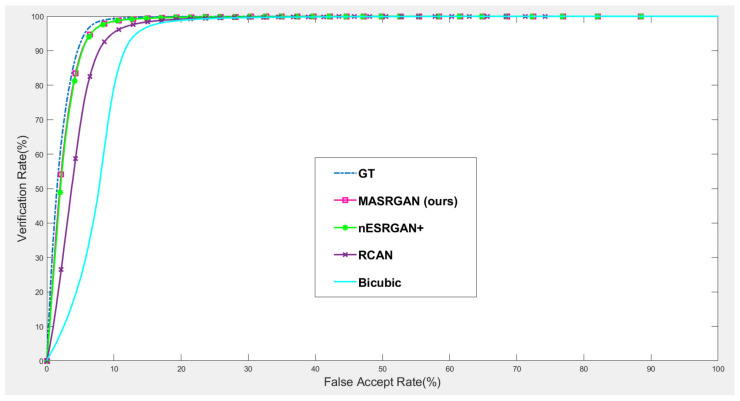
The comparison of the ROC curves of face recognition based on input images enhanced by the various SR models on CelebA dataset.

**Figure 16:**
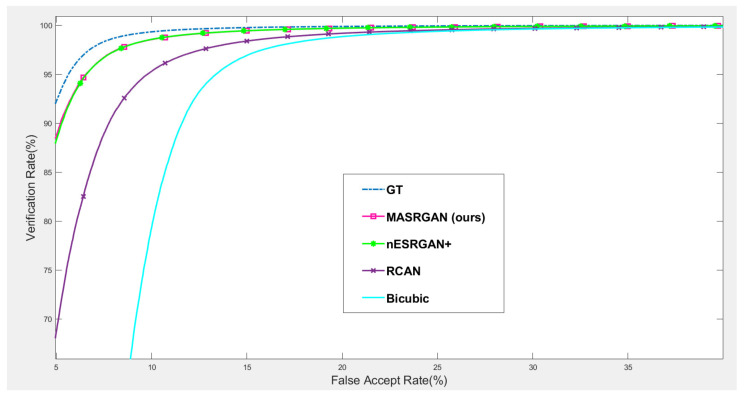
The comparison of ROC curves between nESRGAN+ (current SOTA) and MA-SRGAN (SR/wMsk) on the CelebA dataset.

Our proposed MA-SRGAN can be applied to face recognition and it also works better than most of the SR methods in terms of EER, as Figure 15 describes. In the further comparison, the proposed method achieves higher VR than the nESRGAN+ with the lower FAR. Furthermore, MA-SRGAN achieved an outstanding result in 6.23% of EER, which is lower than the latest SOTA and partially reflects the 89.75% VR, shown in Figure 16 as well as Table 4. This also highlights the benefits of using the attention module, which can also present more informative details about the distinguishable part of face recognition.

#### 4.5.3. Visual Evaluation

The objective metric of biometric recognition has already shown the superiority of the proposed method, while visual evaluation is an alternative to judge the image quality in SR field. Therefore, in this section, we also present several visual comparisons with both the iris images and the face images on the ROI part according to the domain-specific requirement.

**Figure 17:**
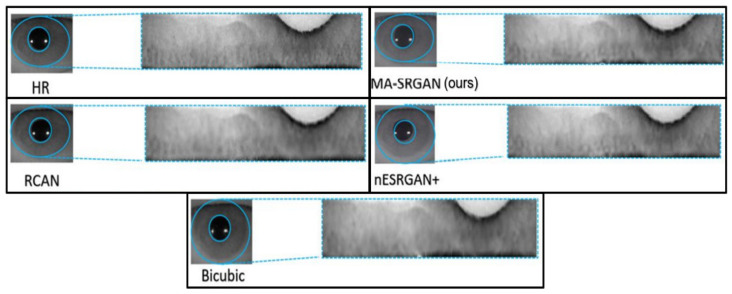
Visual results with Bicubic degradation (×4) on “Rcls6_10” from the CASIA dataset.

**Figure 18:**
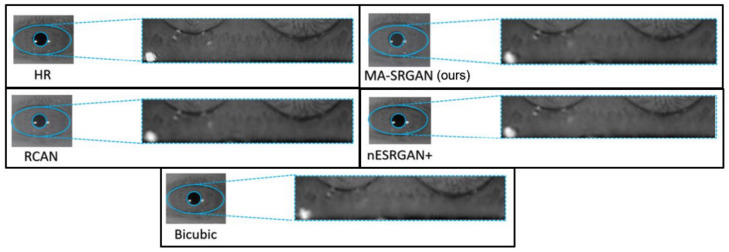
Visual results with Bicubic degradation (×4) on “Rcls489_7” from the CASIA dataset.

We show visual comparisons on normalized iris image, as illustrated in Figure 17 and Figure 18. For both image sets, we observed that most of the compared methods cannot recover the iris texture and would suffer from blurring artifacts. In contrast, our MA-SRGAN can slightly alleviate such side-effect and restore texture to some degree, which in turn helps to enhance the recognition rate.

**Figure 19:**
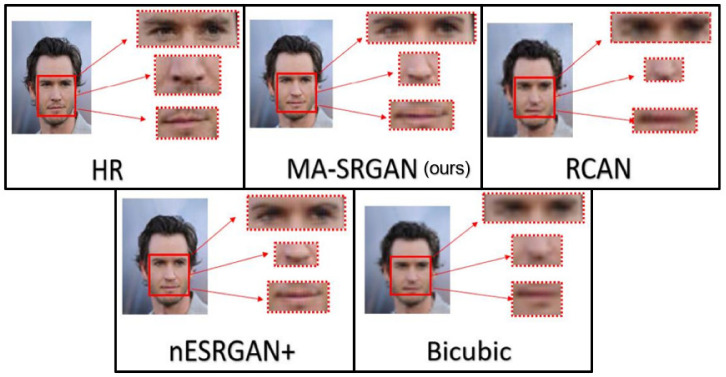
Visual results with Bicubic degradation (×4) on “011006” from the CelebA dataset.

**Figure 20:**
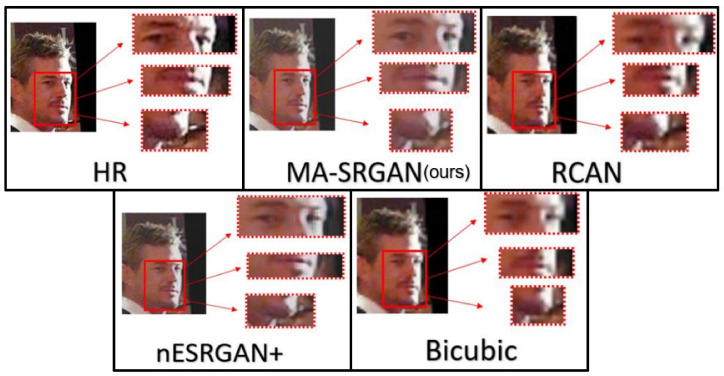
Visual results with Bicubic degradation (×4) on “064049” from the CelebA dataset.

We show visual comparisons for super-resolved face images, highlighting the multi-ROI parts, as illustrated in Figure 19 and Figure 20. For both image sets, we observed that most of the compared methods produces blurring artifacts near the region of facial landmarks. Only our MA-SRGAN produces more faithful results compared to the HR image and it enhances facial features such as eye, nose, and mouth.

### 4.6. Discussion about Practicality of the Proposed Method

For now, we have verified the effectiveness of the ROI mask in the application of biometric recognition via both objective analysis as well as subjective visual comparison. Since the input image may not come with the corresponding mask in the practical application, how to create the masks for input images seems to be an open issue in this work.

Fortunately, there exist a lot of sematic segmentation models [38,39], which are able to predict the corresponding mask for the downstream task. We can train such segmentation models beforehand using the given labeled masks. Note that there also exist some segmentation models for special purposes, which may be more suitable for generating ROI masks. For example, Li et al. proposed the robust model for iris segmentation [40], which can even generate high-quality masks in non-cooperative environments. Besides, some preprocessing algorithm may also offer the ROI information for generating corresponding face masks. For example, we can use the facial landmark detection model [41] to predict an accurate center point of the ROI region, and generate the corresponding mask for face images.

Furthermore, a possible improvement of the proposed method is to force the model to learn the feature-rich region in the image by itself. We may directly apply the metric of the downstream task to train the generator, and the ROI information will be inferenced automatically in the model by applying a back propagation algorithm during the training phase.

## 5. Conclusions

We have proposed MA-SRGAN for image SR. Specifically, the user-defined ROI region will force the generator to focus on the reconstruction of the ROI detail. For propagating such information to the generator, a new extra-part of the discriminator has been introduced to further increase the precision of focusing on the correct part by the given ROI mask, and L1 loss is used to enhance the model robustness against image noise during the training. Moreover, we found that the SR model which takes the domain knowledge into account will contribute more to the downstream task. All these improvements have contributed so that the deep model can learn the correspondence between HR and SR images and is able to generate images with more detailed and discriminative information inside ROI. As a result, the proposed MA-SRGAN outperforms the current SOTA (nESRGAN+) in the task of iris and face recognition by 1.3% verification rate in large-scale biometric experiments.

For future works, we plan to design new modules and force the deep model to learn the ROI automatically during the training process. This will eliminate the need for human labor to denote the ROI and make the proposed method more practical and useful in many scenarios for mobile sensing networks.

## Figures and Tables

**Table 1:** Comparison of spatial sample ratio based on ISO standard.

	Iris Image	Face Image
Standard	ISO/IEC 29794-6	ISO/IEC 19794-5
Spatial Sample Ratio	15.7 pixels/millimeter	120 pixels/inter-eye

**Table 2:** Dataset Specification.

	CASIA	CelebA
Number of class	2000	6000
Number of images in each class	10	20
Image resolution (h × w)	640 × 480	160 × 200

**Table 3:** The comparison with different resolution in terms of EER and corresponding criteria.

	LR	Bicubic	RCAN	nESRGAN+	MASRGAN (Ours)	GT
EER	5.500	3.796	2.430	2.488	**2.308**	2.072
Fisher Ratio	1.535	1.687	1.86	1.852	**1.873**	1.932
VR ^1^	75.62%	84.35%	91.74%	91.65%	**92.23%**	93.62%

^1^ The table presents the Verification Rate for each method along with the alignment of 1% False Accept Rate, and the bold style text indicate the best result with the corresponding metric.

**Table 4:** The comparison with different resolution in terms of EER and corresponding criteria.

	Bicubic	RCAN	nESRGAN+	MASRGAN (Ours)	GT
EER	11.310	8.260	6.247	**6.237**	5.506
Fisher Ratio	1.16	1.40	**1.65**	1.64	1.86
VR ^1^	24.18%	68.74%	87.71%	**89.75%**	92.01%

^1^ The table presents the Verification Rate for each method along with the alignment of 5% False Accept Rate, and the bold style text indicate the best result with the corresponding metric.

## Data Availability

In this study, we use a public iris dataset, CASIA-v4, that can be found here: http://www.cbsr.ia.ac.cn/china/Iris%20Databases%20CH.asp (accessed on 20 July 2021). Besides, we also use a public face dataset, CelebA, that can be found here: https://mmlab.ie.cuhk.edu.hk/projects/CelebA.html (accessed on 3 September 2021).
